# Omentin-1 Is Decreased in Maternal Plasma, Placenta and Adipose Tissue of Women with Pre-Existing Obesity

**DOI:** 10.1371/journal.pone.0042943

**Published:** 2012-08-28

**Authors:** Gillian Barker, Ratana Lim, Harry M. Georgiou, Martha Lappas

**Affiliations:** Department of Obstetrics and Gynaecology, University of Melbourne, Victoria, Australia Mercy Perinatal Research Centre, Mercy Hospital for Women, Heidelberg, Victoria, Australia; University of Las Palmas de Gran Canaria, Spain

## Abstract

**Objective:**

The aim of this study was to determine (i) the effect of maternal obesity and gestational diabetes mellitus (GDM) on (i) the circulating levels of omentin-1 in cord and maternal plasma, and (ii) gene expression and release of omentin-1 from human placenta and adipose tissue. The effect of pregnancy on circulating omentin-1 levels was also determined.

**Design:**

Omentin-1 levels were measured in maternal and cord plasma from obese and non-obese normal glucose tolerant women (NGT; n = 44) and women with GDM (n = 39) at the time of term elective Caesarean section. Placenta and adipose tissue expression and release of omentin-1 was measured from 22 NGT and 22 GDM women collected at the time of term elective Caesarean section. Omentin-1 levels were also measured in maternal plasma from 13 NGT women at 11 and 28 weeks gestation and 7 weeks postpartum.

**Results:**

Maternal obesity was associated with significantly lower omentin-1 levels in maternal plasma; however, there was no effect of maternal obesity on cord omentin levels. Omentin-1 gene expression was lower in placenta and adipose tissue obtained from women with pre-existing obesity. In addition to this, adipose tissue release of omentin-1 was significantly lower from obese pregnant women. Omentin-1 levels were significantly lower in non-obese GDM compared to non-obese NGT women. However, there was no difference in omentin-1 levels between obese NGT and obese GDM women. There was no effect of GDM on cord omentin levels, and placental and adipose tissue omentin-1 expression. Maternal omentin-1 levels were negatively correlated with fetal birthweight and fetal ponderal index.

**Conclusions:**

The data presented in this study demonstrate that pre-existing maternal obesity is associated with lower omentin-1 expression in placenta, adipose tissue and maternal plasma. Alteration in omentin-1 in pregnancy may influence the development of metabolic disorders in offspring later in life.

## Introduction

Gestational diabetes mellitus (GDM) and maternal obesity during pregnancy can affect up to 16–20% of all pregnancies [1], [2], [3], [4]. These metabolic disturbances influence future individual health in both mother and infant [5], [6], [7]. They are associated with increased risk of adverse pregnancy and infant outcomes, and in the long-term, they increase the risk of developing obesity, type 2 diabetes and cardiovascular disease in both the mother and child [6]. In addition to this, they are also associated with substantial societal medical costs [5].

Omentin-1 was first identified from visceral omental adipose tissue in 2003 [8]. There are two highly homologous isoforms of omentin, omentin-1 and omentin-2; however, omentin-1 is the major circulating form in human plasma [9]. It has been linked to obesity [9], [10], [11], type 2 diabetes [10], [12], [13], [14], the metabolic syndrome [15], and polycystic ovary syndrome (PCOS) [16]. Omentin-1 enhances insulin action [17]; is inversely related to obesity [9], [10], [11]; is increased after weight loss [18]; and is downregulated by insulin and glucose [16].

To date, there is a paucity of data on omentin-1 and human pregnancy. The first aim of this study was to determine the effect of pregnancy on maternal omentin-1 levels. The second aim of this study was to determine the maternal and cord serum omentin-1 levels in obese pregnant women and women with GDM. Correlation between omentin-1 levels with insulin resistance and fetal birthweight were also explored. Finally, we sought to elucidate the effect of maternal obesity and GDM on omentin-1 expression and release from placenta and omental adipose tissue.

## Materials and Methods

### Ethics

Written informed consent was obtained from all participating patients. Ethics approval was obtained from the Mercy Hospital for Women's Research and Ethics Committee.

### Study 1 population: Effect of pregnancy on circulating maternal omentin-1 levels

Pregnant women attending the outpatient clinic at the Mercy Hospital for Women (East Melbourne, Australia) were recruited to the study by a clinical research midwife. Blood was collected from all study patients on three separate occasions during the pregnancy: at the first antenatal attendance (approximately 10 weeks gestation); at the time of the oral glucose tolerance test (approximately 28 weeks gestation); and postnatally at the time of a follow-up OGTT (approximately 7 weeks after delivery). Venous blood was collected for routine pathology testing (including glucose determination) and a further 5 ml of blood was collected into a vacuum EDTA tube for research purposes. Blood samples were immediately centrifuged at 1,000 g for 5 min and the plasma aliquoted into 1 ml microfuge tubes and samples were immediately stored at −80°C until assayed for omentin-1 as detailed below. For the purposes of this study, 13 women with an uncomplicated pregnancy were retrospectively selected for blood analysis. This cohort of women has been previously been analysed for various other adipokines [19].

### Study 2 population: Effect of pre-existing maternal obesity and GDM on maternal and cord omentin-1 levels, and placental omentin-1 gene expression

Women were invited to provide samples on the day of admission for surgery. Blood samples used in this study are from participants who were consented during the period 2005–2011. For the tissue explant studies, sample collection occurred over a period of 9 months (2009–2010). Our study population included normal glucose tolerant (NGT) women and women with GDM. All pregnant women were screened for GDM at approximately 28 wks gestation, and women participating in the NGT group had a negative screen. Women with GDM were diagnosed according to the criteria of the Australasian Diabetes in Pregnancy Society (ADIPS) by either a fasting venous plasma glucose level of ≥5.5 mmol/l and/or ≥8.0 mmol/l 2 h after a 75 g oral glucose load. All women that were diagnosed with GDM in this study had a normal glucose tolerance on a 6 week postnatal OGTT.

Women were also stratified according to their BMI (at approximately 10–12 weeks gestation). Lean women were categorised as having a BMI≤24.9 kg/m^2^, overweight patients with a BMI≥25 and ≤29.9, and obese patients with a BMI≥30 kg/m^2^. However, as there was no difference in omentin-1 levels between lean and overweight women, the data from these groups were combined and classified as non-obese (BMI≤29.9).

To control for any effects of labour on endpoint levels, only women who were scheduled for an elective Caesarean section at term (≥37 weeks gestation) were recruited for participation in this study (indications for Caesarean section were breech presentation and/or previous Caesarean section). Women with chorioamnionitis, preeclampsia, preexisting diabetes, asthma, multiple pregnancies, and fetal chromosomal abnormality were excluded.

#### Blood collection and preparation

Maternal and cord blood was collected from 44 NGT and 39 GDM (12 diet-treated and 27 insulin-treated) pregnant women. The clinical characteristics of the subjects in this study are detailed in Table 1. The NGT group consisted of 27 non-obese (18 lean and 9 overweight) and 17 obese pregnant women. Maternal blood was collected by venipuncture approximately 30 minutes prior to the scheduled Caesarean section. Cord blood was collected by needle/syringe from the umbilical vein after delivery. Blood samples were immediately centrifuged at 1,500 *g* for 10 min and the plasma aliquoted into microfuge tubes and samples were immediately stored −80°C until assayed for omentin-1, insulin, leptin, adiponectin and glucose levels as detailed below.

**Table 1:** Plasma study: Characteristics of the study group.

	NGT non-obese (n = 27)	NGT obese (n = 17)	GDM non-obese (n = 21)	GDM obese (n = 18)
**Maternal age (years)**	33.1±0.7	30.8±1.1	35.5±0.8	34.0±1.1
**Maternal BMI at first visit (kg/m^2^)**	23.4±0.6	35.7±1.0*	23.2±0.8	35.6±1.3**
**Maternal BMI at delivery (kg/m^2^)**	27.7±0.7	38.6±1.3*	26.7±0.7	37.8±1.5**
**Gestational age at birth (weeks^+days^)**	39^+0^±0.1	39^+2^±0.2	39^+0^±0.2	39^+2^±0.2
**Fetal Sex**	15 Female; 12 Male	9 Female; 8 Male	11 Female; 9 Male	6 Female; 12 Male
**Fetal birthweight (g)**	3386±68	3628±120	3308±60	3480±107
**Ponderal Index** §	2.5±0.1	2.7±0.1	2.6±0.0	2.7±0.1
**Fasting plasma OGTT (mmol/l)** ‡	4.3±0.1	4.7±0.1*	4.7±0.1*	5.2±0.2** ^†^
**1 hour plasma OGTT (mmol/l)** ‡	7.2±0.4	8.0±0.4	9.2±0.4*	10.0±0.4^†^
**2 hour plasma OGTT (mmol/l)** ‡	5.9±0.3	5.6±0.3	8.9±0.2*	8.1±0.3** ^†^
**Cord insulin at delivery (IU/ml)**	8.8±1.2	8.4±1.2	6.3±0.8	7.3±1.0
**Cord glucose at delivery (mmol/l)**	3.7±0.1	3.7±0.1	3.6±0.2	4.0±0.2
**Cord HOMA-IR at delivery**	1.4±0.2	1.4±0.2	1.0±0.1	1.3±0.2
**Maternal insulin at delivery (IU/ml)**	21.6±2.2	26.6±1.7	24.9±2.2	24.9±2.8
**Maternal glucose at delivery (mmol/l)**	4.0±0.1	4.1±0.2	3.7±0.2	4.3±0.1**
**Maternal HOMA-IR at delivery**	3.8±0.4	4.8±0.4	4.1±0.4	4.8±0.6
**Maternal adiponectin at delivery (µg/ml)**	3.2±0.3	2.4±0.2*	3.8±0.4	3.4±0.5
**Maternal leptin delivery (ng/ml)**	30.7±4.3	46.9±7.9*	20.5±3.0	38.2±8.8
**Cord omentin at delivery (ng/ml)**	58.0±6.0	48.3±9.0	68.4±8.3	58.3±8.6
**Maternal omentin at delivery (ng/ml)**	19.5±2.3	7.1±0.9*	12.1±1.4*	8.2±1.2**

Values represent mean ± SEM.

* *P*<0.05 vs. NGT non-obese;

** *P*<0.05 vs. GDM non-obese.

§ Rohrer's ponderal index is defined as birthweight (g)/birth length^3^ (cm^3^) ×100 [39].

‡ OGTT – oral glucose tolerance test at ∼28 weeks gestation.

#### Tissue preparation and explants

Human placenta and omental adipose tissue were obtained from 22 NGT and 22 GDM (6 diet- and 16 insulin-treated) pregnant women. The clinical details of these patients can be found in [20]. Tissues were obtained within ten minutes of delivery and dissected fragments were placed in ice-cold PBS. Adipose tissue was washed thoroughly in ice-cold PBS to remove any blood, and then cut into 2 mm^2^ pieces. Placental lobules (cotyledons) were obtained from various locations of the placenta; the basal plate and chorionic surface were removed from the cotyledon, and villous tissue was obtained from the middle cross-section. A piece of placenta and adipose tissue (100 mg) was snap frozen in liquid nitrogen and stored at −80°C until required for RNA extraction. The remaining tissue was placed in DMEM (supplemented with penicillin G and streptomycin) at 37°C in a humidified atmosphere of 5% CO_2_ and 8% O_2_ for placenta, and 5% CO_2_ and 21% O_2_ for adipose tissue for 1 h. Explants were blotted dry on sterile filter paper and transferred to 24 well tissue culture plates (100 mg wet weight/well). The explants were incubated in 2 ml DMEM in a humidified atmosphere of 5% CO_2_ and 8% O_2_ for placenta, and 5% CO_2_ and 21% O_2_ for adipose tissue for 24 h. Following 24 h incubation, tissues were collected and assayed for total protein, while the incubation medium was collected and assayed for omentin-1 concentration by ELISA. All data were corrected for total protein and expressed as pg per mg protein. The protein content of tissue homogenates was determined using BCA protein assay (Pierce, Rockford, USA), with BSA as a reference standard, as previously described [21], [22], [23].

### Immunohistochemistry

Immunohistochemistry, performed according to our previously published methods [24], was used to determine the localisation of omentin-1 in human placenta. Mouse monoclonal anti-omentin-1 used at 10 µg/ml was purchased from Abnova (Taipei, Taiwan). Negative control slides, where primary antibody was replaced with normal mouse IgG serum were also included.

### Omentin-1 ELISA

A specific and sensitive enzyme linked immunoassay was used to determine concentration of omentin-1 in maternal and cord plasma, and in tissue explant incubation medium. The omentin-1 ELISA kit was obtained from Cusabio Biotech Co. and performed according to the manufacturers' instructions. Plates were read at 450 nm using a Bio-Rad microplate reader (iMark™ Microplate Absorbance Reader, Bio-Rad Laboratories, Hercules, CA, USA). The calculated interassay and intraassay coefficients of variation (CV) were all less than 10%. The limit of detection of the assay was 1.6 pg/ml.

### Insulin, glucose, leptin and adiponectin assays

Blood glucose determination was performed by the hospital pathology department using an automated glucose oxidase/oxygen-rate method. Standard ELISA assay kits for insulin (Diagnostic Systems Laboratories, Webster, TX; limit of detection 0.26 lU/ml), leptin (Rocky Hill, NJ; limit of detection 63 pg/ml), and adiponectin (R&D Systems, Minneapolis, MN; limit of detection 32 pg/ml), were purchased and used according to the manufacturer's instructions. Insulin resistance at delivery was calculated using the homeostasis model assessment for insulin resistance (HOMA-IR) method where HOMA-IR = fasting plasma glucose (mmol/l) times fasting plasma insulin (µU/ml) divided by 22.5 [25].

### RNA extraction and quantitative RT-PCR (qRT-PCR)

Total RNA was extracted from tissues and cells using Tri Reagent according to manufacturer's instructions (Sigma-Aldrich, Saint Louis Missouri). RNA concentrations were quantified using a spectrophotometer (Smart Spec, Bio-Rad), and RNA quality and integrity was determined via the A_260_/A_280_ ratio. For tissues and cells, one µg of RNA was converted to cDNA using SuperScript® VILO™ cDNA synthesis kit (Invitrogen, Carlsbad, California, USA) according to the manufacturer's instructions. The cDNA was diluted ten-fold, and 2 µl of cDNA was used to perform RT-PCR using Sensimix Plus SYBR green (Quantace, Alexandria, NSW, Australia) and 100 nM of pre-designed and validated primer (QuantiTect primer assays, Qiagen, Germantown, Maryland, USA). The specificity of the product was assessed from melting curve analysis. RNA without reverse transcriptase during cDNA synthesis as well as PCR reactions using water instead of template showed no amplification. Average gene C_T_ values were normalised to the average GAPDH C_T_ values of the same cDNA sample. Fold differences were determined using the comparative C_T_ method.

### Statistical Analysis

Statistical analyses were performed with SPSS software (SPSS for Windows, Version 19, SPSS, Chicago, Illinois, USA). Two sample comparisons were analysed by either a paired sample comparison or Student's t-test. For all other comparisons, analysis was performed using a one-way ANOVA (using Tukey HSD correction to discriminate among the means); homogeneity of data were assessed by Bartlett's test, and when significant, data were logarithmically transformed before further analysis. Linear regression analysis was used to compare maternal and cord omentin-1 levels with variables of interest. To determine which variables were independently associated with maternal or cord blood omentin-1 levels, multiple regression analysis was used. All omentin-1 regression analyses were performed on logarithmically transformed data. Statistical difference was indicated by a *P* value of less than 0.05. Data are expressed as mean ± standard error of the mean (SEM).

## Results

### Effect of human pregnancy on maternal omentin-1 levels

**Figure 1:**
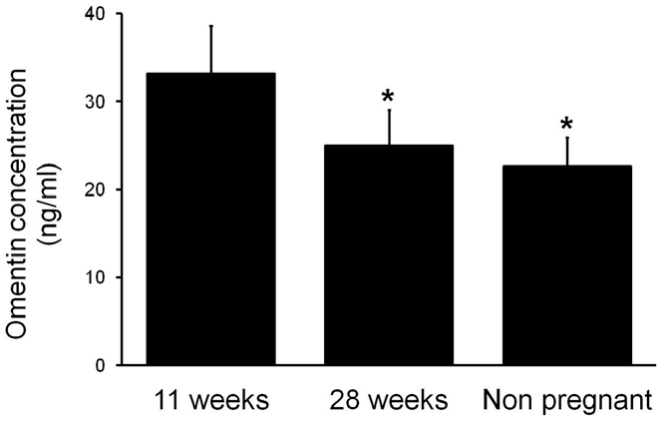
Effect of pregnancy on circulating omentin-1 levels. Omentin-1 levels in women at 11 and 28 weeks gestation and 7 weeks postpartum (non pregnant). Each bar represents the mean ± SEM (n = 11 per group). *P<0.05 vs. 11 weeks gestation.

To determine the effect of human pregnancy on omentin-1 levels, plasma was obtained from 13 NGT subjects at approximately 11 and 28 weeks gestation and 7 weeks postpartum (non pregnant). As shown in Figure 1, omentin-1 levels were higher at 11 weeks gestation compared to both 28 weeks gestation and compared to the non-pregnant state. There was however, no difference in omentin-1 levels between 28 weeks gestation and after pregnancy (7 weeks postnatal).

### Effect of pre-existing maternal obesity on omentin-1 levels during pregnancy in normal glucose tolerant women

#### Maternal plasma and cord plasma-1 omentin-1 levels

Table 1 demonstrates the effect of maternal obesity on maternal and cord omentin-1 levels. There was no difference in omentin-1 levels between lean and overweight patients for both maternal and cord plasma (data not shown), and thus the data combined and referred to as non-obese. In the NGT women, circulating omentin-1 levels were significantly lower in maternal plasma obtained from women with pre-existing obesity. Maternal plasma omentin-1 levels were also significantly lower in non-obese women with GDM compared to non-obese NGT women.

**Figure 2:**
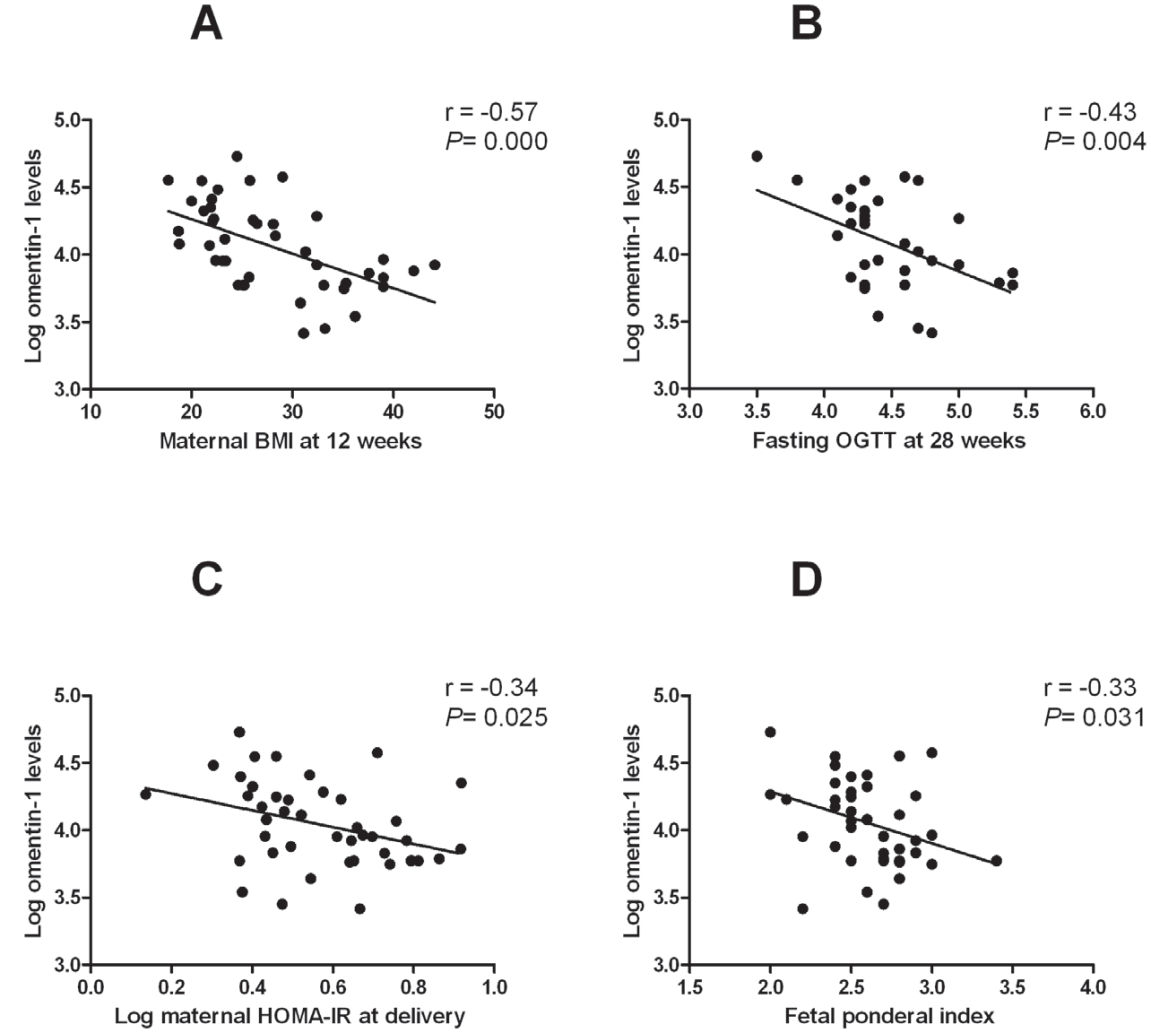
Relationship between maternal omentin-1 levels and clinical features. Scatter plots depicting the relationship between log maternal omentin 1 versus (**A**) maternal BMI at 12 weeks, (**B**) fasting OGTT at 28 weeks, (**C**) log maternal HOMA-IR at delivery and (**D**) fetal ponderal index.

There were significant negative associations between maternal omentin-1 levels and maternal BMI, fasting OGTT, one hour OGTT, maternal insulin levels, maternal HOMA-IR, and fetal ponderal index (Table 2 and Figure 2). There were no associations between maternal omentin-1 levels and maternal leptin or adiponectin levels (data not shown). When multiple regression analysis was performed to determine which variables were independently associated with maternal omentin-1 levels, maternal BMI and fasting OGTT remained significant (Table 3). There were no association between cord omentin-1 levels and any of the parameters studied (data not shown).

**Table 2:** Spearman's rank order correlation analysis.

Log maternal omentin 1 versus:	Correlation Coefficient	*P* value
Maternal BMI at 12 weeks	−0.57	0.000
Log maternal BMI at delivery	−0.43	0.004
Fasting OGTT at 28 weeks	−0.46	0.006
1 hour OGTT at 28 weeks	−0.36	0.033
Log maternal insulin at delivery	−0.32	0.034
Log maternal HOMA-IR at delivery	−0.34	0.025
Fetal ponderal index	−0.33	0.031

**Table 3:** Multiple linear regression analysis.

Log maternal omentin 1 versus:	Correlation Coefficient (beta)	P value
Maternal BMI at 12 weeks	−0.98	0.001
Log maternal BMI at delivery	−0.56	0.05
Fasting OGTT at 28 weeks	−0.28	0.09
1 hour OGTT at 28 weeks	−0.08	NS
Fetal ponderal index	−0.10	NS

NS, not significant.

#### Placenta and maternal adipose tissue omentin-1 expression and release

**Figure 3:**
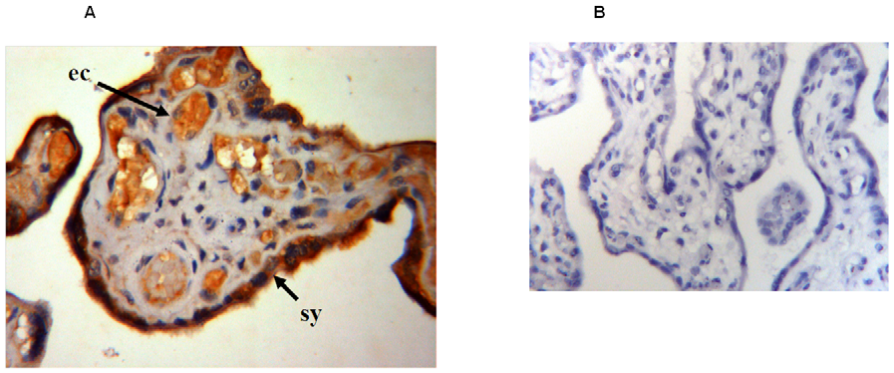
Localisation of omentin-1 in human placenta. (**A**) Immunohistochemical localisation of omentin-1 protein in human placenta. Omentin-1 staining was mainly contained in syncytiotrophoblasts (sy) and endothelial cells (ec). Magnification 400×. (**B**) No specific staining is seen in the negative control for placenta. Magnification 250×.

Omentin-1 is expressed in stromal and vascular cells in adipose tissue [17]; however, the placental location of omentin-1 has not been described. Immunohistochemistry for omentin-1 demonstrated that it was localised mainly in the syncytiotrophoblast layer and endothelial cells (Figure 3).

**Figure 4:**
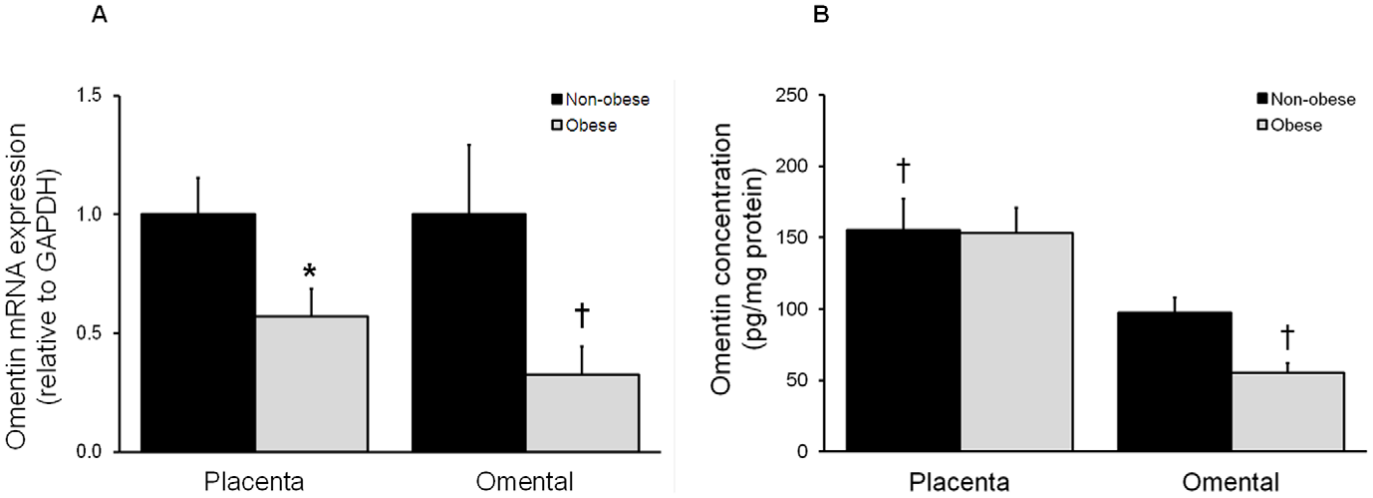
Effect of obesity on omentin-1 expression and release from placenta and omental adipose tissue. Omentin-1 (**A**) gene expression and (**B**) release from human placenta and omental adipose tissue obtained from NGT non-obese (n = 14 for placenta and = 13 for omental) and obese (n = 8) pregnant women. Each bar represents the mean ± SEM. *P<0.05 vs. non-obese placenta; ^†^ *P*<0.05 vs. non-obese adipose tissue.

The effect of maternal obesity on omentin-1 expression and release from placenta and adipose tissue is presented in Figure 4. The release of omentin-1 was greater in placenta compared to adipose tissue. The gene expression of omentin-1 was significantly lower in placenta and adipose tissue obtained from women with pre-existing maternal obesity (Figure 4A). Similarly, adipose tissue from obese pregnant women released significantly less omentin-1 than that from non-obese women (Figure 4B). There was, however no effect of maternal obesity on omentin-1 release from placenta.

### Effect of gestational diabetes on omentin-1 levels during pregnancy

#### Maternal plasma and cord plasma-1 omentin-1 levels

Demographic data of the NGT and GDM participants involved in this investigation are summarised in Table 1. Given that pre-existing maternal obesity was associated with lower omentin-1 levels, we stratified the women in this study according to BMI as either non-obese or obese. There were no significant differences in maternal age, gestational age at delivery, fetal birthweight, fetal ponderal index and cord and maternal plasma glucose and insulin levels at delivery between the NGT and GDM. Table 1 also demonstrates the effect of GDM on maternal and cord omentin-1 levels. Maternal plasma omentin-1 levels were significantly lower in non-obese women with GDM compared to non-obese NGT women. On the other hand, no differences between NGT and GDM omentin-1 levels were detected in the obese cohort. There was no effect of maternal obesity or GDM on cord plasma omentin-1 levels. In addition, there was no significant difference in maternal and cord omentin-1 levels between women with GDM who were managed by dietary modification alone compared to women who were treated with insulin (data not shown).

#### Placenta and maternal adipose tissue omentin-1 expression and release

**Figure 5:**
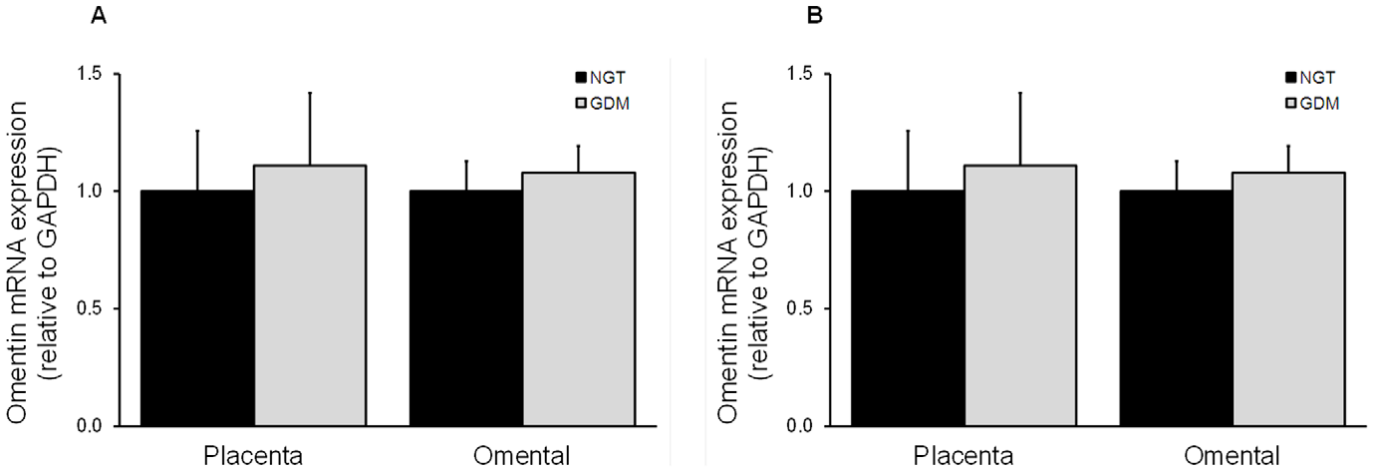
Effect of GDM on omentin-1 expression and release from placenta and omental adipose tissue. Omentin-1 (**A**) gene expression and (**B**) release from human placenta and omental adipose tissue from NGT (n = 22 for placenta; n = 21 for omental and subcutaneous adipose tissue) and GDM (n = 22 for placenta; n = 20 for omental adipose tissue; n = 19 for subcutaneous adipose tissue) pregnant women. Each bar represents the mean ± SEM.

As shown in Figure 5, there was no effect of GDM on placental and adipose tissue omentin-1 gene expression or release.

## Discussion

The main findings of this study are that during maternal obesity in pregnancy is associated with decreased circulating omentin-1 in maternal plasma, and reduced adipose tissue and placenta mRNA expression of omentin-1. Additionally, in non-obese women, maternal omentin-1 levels are significantly lower in GDM women compared to NGT women. On the other hand, in obese women, there is no difference in maternal omentin-1 levels between GDM and NGT women. There was also no effect of GDM on cord plasma omentin-1 levels, nor on its expression in placenta and adipose tissue. Maternal plasma omentin-1 levels were inversely related to measures of insulin sensitivity, including 28 week fasting and one hour OGTT, and insulin levels and HOMA-IR at delivery. Interestingly, there was also a negative relationship between maternal omentin-1 levels and fetal ponderal index.

Decreased omentin-1 has been observed in type 2 diabetes [10], [12], [13], [14] and the metabolic syndrome [15]. In support of these findings, there was decreased omentin-1 levels in maternal plasma of non-obese GDM women compared to non-obese NGT women. On the other hand, in the obese population, there was no difference in omentin-1 levels between NGT and GDM women. Additionally, there was no difference in placental and maternal adipose tissue omentin mRNA expression and release between NGT and GDM women. The only other study in GDM [26] reported no difference in omentin-1 levels in maternal and cord plasma from NGT and GDM women. In omental adipose tissue explants, glucose and insulin significantly dose-dependently decreases omentin-1 mRNA expression and secretion into conditioned media [16]. However, in this study, there was no difference in omentin-1 levels between GDM women treated by diet or insulin.

In this study, significant negative linear relationships were observed between maternal omentin-1 levels and maternal BMI, maternal insulin and HOMA-IR at delivery as well as 28 week fasting and one hour OGTT. However, multiple linear regression analysis revealed significant negative associations between maternal omentin-1 levels and maternal BMI and fasting OGTT. Other studies have reported similar observations; circulating omentin-1 concentrations correlate negatively with BMI, fasting insulin, and homeostasis model assessment [9], [14], [27]. Of note, there were no significant associations between maternal omentin-1 levels and maternal leptin or adiponectin levels at delivery. Notwithstanding, this is the first study showing a direct association between circulating omentin-1 and markers of insulin sensitivity in pregnant subjects.

Several reports have shown that circulating omentin in serum reflects its expression in visceral adipose tissue, which decreases in parallel with the increase of visceral obesity [11], [16], [17]. This study confirmed these observations. That is, maternal omental adipose tissue from obese women is characterised by significantly lower expression and release of omentin-1. Omentin-1 expression in placenta was also significantly lower in obese women than in controls. To the best of our knowledge, this is the first study in humans describing an association between maternal obesity and placental omentin-1 expression. Although the gene expression of omentin-1 was lower in placentas from obese women, there was no difference in placental omentin-1 release between lean and obese women. Nevertheless, this is the first report showing that human placenta secretes significant amounts of omentin-1. In fact, the release of omentin-1 was significantly higher from placenta than from omental adipose tissue.

It has recently been reported that omentin-1 is present in the fetus and neonate, but it not associated with fetal growth restriction [28]. Similarly, in this study, no correlations between cord omentin-1 levels and birthweight or ponderal index were detected. On the other hand, maternal omentin-1 levels were negatively associated with fetal ponderal index. This finding is not entirely surprising given that the obese women in this study delivered larger babies.

In this study, we report that maternal omentin-1 levels are higher in the first trimester (approximately 11 weeks gestation) compared to both the second trimester (approximately 28 weeks gestation) and the non-pregnant state. There was however, no difference in omentin-1 levels between 28 weeks gestation and after pregnancy. Several explanations may account for this finding. Placenta is the major source of omentin-1 levels. However, for this to be the case, there must be increased omentin-1 clearance in the later stages of pregnancy. It may also be due differences in the secretion of omentin-1 from maternal adipose tissue. Higher omentin-1 in the first trimester of pregnancy may be due to increased fat accretion [29] or reduced secretion from maternal adipose tissue later in pregnancy. Further studies are required to determine if there is increased omentin-1 clearance in the later stages of pregnancy or reduced secretion from maternal adipose tissue. Of note, the non-pregnant samples were taken from women approximately 7 weeks post partum, and breastfeeding may alter omentin-1 levels. Unfortunately, we do not know how many women in study 1 were breast feeding. However, we did test an independent set of samples from non pregnant women whose last pregnancy was least 2 years ago. The values of omentin-1 in these women were not different from those reported in this study (data not shown), suggesting that breastfeeding may not affect omentin-1 levels.

The biological function(s) of omentin-1 in human pregnancy is not known, but it may have a role in regulating blood glucose levels; it enhances insulin-stimulated glucose uptake in human subcutaneous and visceral adipocytes [17]. The glucose transporter, GLUT-4, plays a major role in insulin-stimulated glucose uptake [30]. Previous studies from our laboratory have shown decreased GLUT-4 expression in both placenta [31] and adipose tissue [32] from obese pregnant women. Further studies are required to determine if omentin-1 regulates glucose uptake in adipose tissue and/or placenta of women with obesity.

Omentin-1 may play a role in the development of vascular disease associated with obesity. Omentin-1 expression is lower in patients with carotid atherosclerosis [15], omentin-1 is negatively associated with endothelial dysfunction [27], and omentin-1 serum concentrations are lower in patients with acute coronary artery disease [33]. Whether omentin-1 is related to vascular dysfunction in this cohort of patients is not known; however, obese pregnant women have subclinical atherosclerosis, vascular dysfunction and low-grade inflammation [34], [35]. Omentin-1 has also been shown to inhibit TNF-α induced vascular inflammation in human endothelial cells [36]. Collectively, these observations suggests a protective role against the development of vascular disease. Normal placental angiogenesis ensures adequate placental blood flow to support normal fetal growth [37]. Maternal obesity also has adverse effects on the feto-placental vasculature [38]. Thus, the lower placental omentin-1 levels observed in this study in association with maternal obesity may have long-term implications for fetal development and growth.

In conclusion, novel data is presented in this paper of decreased plasma omentin-1 levels as well as decreased expression of omentin-1 levels in placenta and adipose tissue of women with obesity. Although the physiologic and pathologic significance of these findings remain to be elucidated, it may indicate a mechanism for the development of insulin resistance or vascular disease in obese pregnant women and their offspring.
